# Extraneurological Presentations of Tick‐Borne Encephalitis Virus: A Rare Case of TBEV‐Associated Myocarditis With Fever and Bicytopenia and a Systematic Literature Review

**DOI:** 10.1155/crdi/3051689

**Published:** 2026-02-20

**Authors:** Marco Seneghini, Fabiola Lugano, Micha T. Maeder, Regine Garcia-Boy, Carol Strahm

**Affiliations:** ^1^ Departement of Infectious Diseases and Hospital Hygiene, Cantonal Hospital of St. Gallen, St. Gallen, Switzerland, kssg.ch; ^2^ Department of Internal Medicine, Cantonal Hospital of St. Gallen, St. Gallen, Switzerland, kssg.ch; ^3^ Department of Cardiology, Cantonal Hospital of St. Gallen, St. Gallen, Switzerland, kssg.ch; ^4^ Division of Human Microbiology, Center for Laboratory Medicine, St. Gallen, Switzerland

**Keywords:** myocarditis, myopericarditis, TBE, TBEV, tick-borne encephalitis, tick-borne encephalitis virus, ticks

## Abstract

**Purpose:**

Tick‐borne encephalitis (TBE), a zoonotic disease caused by the tick‐borne encephalitis virus (TBEV), usually manifests with a biphasic course with neurological involvement during its second phase. Extraneurological manifestations are rare but clinically relevant.

**Methods:**

We report a case of a 77‐year‐old patient with suspected acute myocarditis associated with central European TBEV infection and summarize the literature on extraneurological TBEV manifestations through a systematic review.

**Results:**

The case of a 77‐year‐old patient with suspected acute myocarditis associated with central European TBEV infection is reported and discussed. Literature analysis identified 23 publications reporting extraneurological TBEV manifestations, including (peri)myocarditis, myositis, hematological abnormalities, and elevated transaminases.

**Conclusion:**

TBEV infection can be complicated by myocarditis and should therefore be considered in the differential diagnosis in areas where TBEV is endemic in a patient with risk factors for tick bites. Vaccination remains crucial for preventing TBEV infections.

## 1. Introduction

Tick‐borne encephalitis (TBE) is a viral disease transmitted by ticks, caused by the tick‐borne encephalitis virus (TBEV). According to the current International Committee on Taxonomy of Viruses (ICTV) classification (MSL38), TBEV is a member of the species *Orthoflavivirus encephalitidis* within the genus *Orthoflavivirus*, family Flaviviridae [1]. TBEV infections are endemic in numerous countries across the Northern Hemisphere, spanning Europe and Asia. TBEV is characterized by pronounced genetic diversity and is traditionally divided into three major subtypes—European (TBEV‐Eur), Siberian (TBEV‐Sib), and Far Eastern (TBEV‐FE)—with additional lineages, including Baikalian and Himalayan variants, now recognized [2, 3]. The geographic distribution of the TBEV complex appears to be wide, and overlap of the TBEV complex with other viruses was observed in some areas [4]. For the scope of our study, we have chosen not to differentiate between the three lineages and collectively refer to them as TBEV. This approach simplifies our analysis and allows us to explore the broader implications of extraneurological manifestations of TBEV without delving into the nuances of lineage‐specific differences.

Transmission occurs mainly through the bite of infected *Ixodes* species ticks, acting as vectors. Much like other flaviviruses, the majority of TBEV infections remain asymptomatic. However, when symptoms do emerge, the infection typically unfolds as a biphasic febrile illness. In the initial phase, affected individuals often experience flu‐like symptoms, encompassing fever, headache, muscle aches, and fatigue [5]. As the disease progresses, the second phase unfolds, characterized by the involvement of the central nervous system (CNS), which gives rise to the most severe form of TBE [6]. Even though extraneurological manifestations of TBEV infections are infrequent, they have been reported, including hepatitis [7], myositis [8], myocardial involvement [9], thrombocytopenia, and leukopenia [10].

Given their infrequent occurrence, the presence of isolated extraneurological manifestations presumably during the first phase of the illness potentially leads to challenges in recognizing TBEV as the underlying cause of the patients’ symptoms and hence unnecessary diagnostic procedures. To highlight this diagnostic challenge and contribute to the growing body of knowledge on TBEV’s extraneurological manifestations, we present the case of a 77‐year‐old man with perimyocarditis most likely due to TBEV. Furthermore, we aim to provide a comprehensive systematic review of the available evidence on this topic, synthesizing current understanding and identifying areas for future research.

## 2. Materials and Methods

**FIGURE 1:**
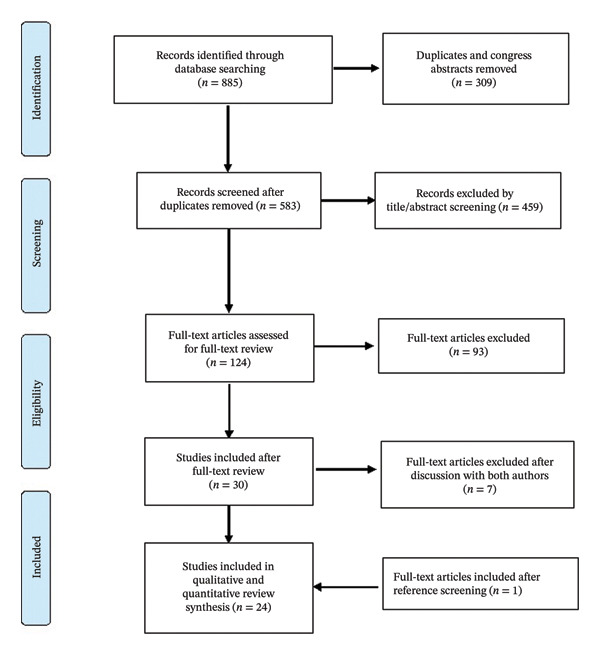
PRISMA flow diagram of the study selection process for the systematic review of extraneurological manifestations of tick‐borne encephalitis virus infection.

We describe a case of a 77‐year‐old man with perimyocarditis most likely due to TBEV infection. We performed a systematic literature search to identify articles related to extraneurological manifestations of TBE/TBEV infections. Additionally, we developed a protocol for a systematic review that was registered with PROSPERO (CRD42024534277) [11] and followed the PRISMA guidelines. We developed a search strategy in PubMed and then adapted it for Embase and Cochrane databases. The strategy included terms related to TBE/TBEV and various extraneurological manifestations. The search strategy used in these databases were [“Tick‐borne Encephalitis” OR “TBE” OR “TBE Virus” OR “TBEV” OR “Tick‐borne encephalitis Virus” OR “Tick‐borne meningoencephalitis” OR “FSME” OR “Frühsommer‐meningoenzephalitis” OR “FSME‐virus”] AND [“Extracerebral” OR “Extra‐cerebral” OR “extra‐CNS” OR “Extra‐neurological” OR “extraneurological” OR “Atypical” OR “Myocarditis” OR “Perimyocarditis” OR “Cardiac” OR “Respiratory” OR “Muskuloskelettal” OR “Hepatitis” OR “Gastrointestinal” OR “Myositis” OR “Hematological” OR “Leukopenia” OR “Thrombocytopenia” OR “Agranulocytosis” OR “Pancytopenia”]. We identified 23 articles regarding extraneurological manifestations of TBE/TBEV infections. Our own case report was added to these 23 articles, bringing the total number of studies included in the qualitative and quantitative review synthesis to 24, as shown in Figure 1. Articles were selected for inclusion if they represented original work relating to patients with confirmed TBE/TBEV infection and extraneurological manifestations, including letters to the editor, case reports, case series, and observational studies. Article selection was performed by two independent reviewers. Relevant articles were screened for additional references. Data disparities were resolved by discussion and mutual agreement among the reviewers.

## 3. Results

### 3.1. Case Presentation

A 77‐year‐old man presented to the emergency department (ED) with acute chest pain, radiating to the back which led to admission due to suspected acute coronary syndrome. He had no known cardiac disease and no prior episodes of chest pain. Three weeks prior to presentation, he had sustained a tick bite. In the ED, the patient was febrile but in no apparent distress. Ear temperature was 38.5°C, heart rate was regular at 62 beats per minute, blood pressure was 141/70 mmHg, and oxygen saturation was 94% while breathing ambient air. There were no neurological deficits.

At admission, laboratory results (Table 1) were significant for a moderate leukopenia (1.9 G/L, normal value [NV] 4–10 G/L), severe neutropenia (neutrophil count 0.3 G/L, NV 2–8 G/L), and thrombocytopenia (102 G/L, NV 150–300 G/L). Cardiac biomarkers at admission were only slightly increased, with a high sensitivity troponin I of 19.1 ng/L (NV < 18 ng/L) and a normal creatine kinase (CK) (85 U/L, NV < 170 U/L). Additional blood analyses were significant for an increase in hepatic enzymes (aspartate aminotransferase [AST] of 133 U/L, NV < 55 U/L; alanine aminotransferase [ALT] of 109 U/L, NV < 55 U/L; and an elevated ferritin level of 1824 μg/L, NV 30–330 μg/L). Blood cultures showed no bacterial growth. Nasopharyngeal swab for influenza A, B, respiratory syncytial virus, adenovirus, SARS‐CoV‐2, and enterovirus by PCR was negative. For the further course of laboratory values, see Table 1. The initial electrocardiogram showed a mild bradycardic sinus rhythm (52 bpm) without ST/T wave abnormalities. The chest radiograph was normal. Transthoracic echocardiography revealed preserved left ventricular function (LVEF 65%) without regional wall motility or pericardial effusion. A coronary angiography showed normal coronary arteries.

**TABLE 1:** Laboratory values.

Investigation	Reference range	Admission	Day 7	Day 11	Day 70
White cell count (G/L)	4.0–10.0	3.8	1.9	2.9	4.2
Neutrophilic granulocytes (G/L)	1.6–7.4	0.3	0.4	1.0	2.4
Platelet count (G/L)	150–300	150	102	144	211
Aspartate aminotransferase (U/L)	< 55	133	133	101	24
Alanine aminotransferase (U/L)	< 55 U/L	109	109	113	19
Hs troponin I (ng/L)	< 18	19.1	34.7	39	
Creatine kinase (U/L)	< 170	85	401	123	
TBE‐virus IgM (U/mL)	< 10 U/mL		63		148
TBE‐virus IgG (U/mL)	< 100 U/mL		< 30		> 3000

An early (i.e., first phase) TBEV infection was suspected based on the presence of a febrile illness with a concomitant history of a tick bite, and blood analysis for tick‐borne infections was sent for testing. TBEV serology returned positive (IgM elevated with subsequent seroconversion to high‐titer IgG), consistent with acute infection. Despite residing in an area recognized as endemic for TBEV in Eastern Switzerland, the patient had never received prior immunization against the virus.

Serologic testing showed positive antibodies directed against TBEV (IgM 63 U/mL, IgG < 30 U/mL), consistent with acute infection. Given the constellation of typical chest pain, rise in cardiac biomarkers, inflammatory laboratory abnormalities, and exclusion of coronary disease, the diagnosis of clinically suspected acute myocarditis was made according to consensus criteria [12]. Comprehensive investigations were undertaken to rule out other tick‐borne diseases and potential myocarditis‐related infections, as detailed in Table 2. Given the patient’s fever‐free progression, alongside modest elevation of cardiac markers with limited dynamics and clinical improvement, the decision was made to forego further assessment via cardiovascular magnetic resonance imaging (cardiac MRI) or myocardial biopsy. The patient recovered rapidly and was successfully discharged within 14 days. Subsequently, during a follow‐up consultation held 10 weeks post‐discharge, the patient’s overall health exhibited significant improvement, although there were occurrences of two episodes of intense frontal headaches and night sweats. Importantly, laboratory values had returned to within the normal range. A significant seroconversion for TBEV was verified, characterized by IgM levels at 148 U/mL (normal: ≤ 10 U/mL) and IgG levels surpassing 3000 U/mL (normal: ≤ 100 U/mL).

**TABLE 2:** Summary of serologic investigation results.

**Investigation**	**Reference range**				
**Day from hospitalization**		**Day 1**	**Day 7**	**Day 11**	**Day 70**

IgM against Rickettsia rickettsia/conorii	< 1:64				< 1:64
IgG against Rickettsia rickettsia/conorii	< 1:64				< 1:64
IgM against parvovirus B19	< 0.9 index				< 0.10
IgG against parvovirus B19	< 0.9 Index				9.5
HIV‐1/2 Ak/Ag			negative		
Coxiella burnetii Phase II IgM	< 0.9 MOC				0.3
Coxiella burnetii Phase II IgG	< 20 U/mL				22.7
Coxiella burnetii Phase I IgG	< 0.9 MOC				0.2
Coxiella burnetii Phase I IgG	< 0.9 MOC				0.1
IgM against Francisella tularensis	< 10 U/mL				< 4
IgG against Francisella tularensis	< 10 U/mL				< 3
IgM against Bartonella quintana	< 1:20				neg
IgG against Bartonella quintana	< 1:64				1:128
IgM against Ehrlichia	< 1:20				< 1:20
IgG against Ehrlichia	< 1:64				< 1:64
IgM against Borrelia burgdorferi	< 18 AU/mL				< 5
IgG against Borrelia burgdorferi	< 10 AU/mL				4
IgM against TBE	< 10 U/mL		< 30		148
IgG against TBE	< 100 U/mL		63		> 3000

## 4. Discussion

Our case sheds light on an intriguing aspect of TBEV infection, showing bicytopenia, mild transaminase elevation, and clinical features of myocarditis without neurological involvement. To contextualize this case, a systematic review was conducted following a registered protocol (PROSPERO CRD42024534277). PubMed, Embase, and Cochrane searches using terms for TBEV and extraneurological manifestations identified 23 relevant studies. Reports described cardiac, muscular, hepatic, and hematological involvement during TBEV infection. A quantitative summary of extraneurological manifestations reported in the literature, including cardiovascular, muscular, hepatic, and hematological involvement, is provided in Table 3.

**TABLE 3:** Pooled prevalence of extraneurological clinical features and laboratory findings of patients with TBE infection in the present systematic review.

Disorder	Studies (*n*)	Patients (*n*)	Comments
(Peri)myocarditis	3	7	
Myositis	5	5	Elevated creatine kinase above the upper limit of normal with muscle‐related symptoms
Hepatic manifestations			
Elevated transaminases, all	11	112	At least one abnormal transaminase value
Elevated transaminases ≥ 5 upper limit	5	21	
Elevated cholestatic enzymes	2	2	Elevated alkaline phosphatase with or without bilirubin and/or GGT elevation
Hematologic manifestations			
Leukopenia (WBC < 4.0 × 10^9^/L)	11	183	
Thrombocytopenia (PC < 150,000/μm^3)^	8	98	
Neutropenia (ANC < 1.5 × 10^9^/L)	2	91	
Agranulocytosis (ANC < 0.1 × 10^9^/L)	1	1	
Hemophagocytic lymphohistiocytosis (HLH)	1	1	Immunosuppressed 12‐year‐old patient
Hemorrhagic syndrome	1	8	Associated with Siberian/Far Eastern TBEV subtypes
Other manifestations			
Exanthema	2	2	
Diarrhea	2	2	

Myocarditis: Our investigation revealed three studies involving a total of seven patients with myocarditis or perimyocarditis as an extraneurological manifestation of TBEV. Duppenthaler et al. [9] documented the case of an 11‐year‐old girl with TBEV‐associated perimyocarditis who presented with fever, severe headache, vertigo, photophobia, confusion, and abdominal pain. Echocardiography revealed pericardial effusion, along with elevated inflammatory markers and cardiac troponin. A comparative study conducted by Tesarova‐Magrova and Kroo [13] in 1966 examined clinical, laboratory, and electrocardiographic findings from 53 patients with acute TBE and 53 patients with non‐TBE encephalitis. This study indicated potential myocarditis in five TBE cases and three non‐TBE cases. Similarly, Hofbauer [14] described the case of a 55‐year‐old patient with TBE with postulated myocarditis based solely on the patient’s electrocardiographic abnormalities consistent with myocardial damage and with complete clinical recovery.

Myositis: In our literature analysis, we identified five distinct cases [8, 15–18] associated with myositis, characterized by the inflammation of skeletal muscles, accompanied by an increase in CK levels and manifesting with localized or diffuse pain, swelling, and/or weakness. Notably, none of these cases exhibited compartment syndrome. Among these instances, three of them [8, 16, 17] had a favorable clinical outcome. However, it is important to note that the case from Montyvidaite et al. [16] exhibited a particularly severe clinical course requiring intensive care, albeit ultimately achieving complete clinical recovery. In the case from Popović Dragonjić [15], involving a 58‐year‐old male with a fatal TBE, the authors postulated that the elevated CK levels might have been attributable to TBE‐associated muscle inflammation consistent with myositis. In contrast, the case reported by Zambito Marsala et al. [18], involving a 60‐year‐old man, a poliomyelitis‐like paralysis emerged as the sole clinical manifestation of TBE, coupled by initially severe isolated hyperCKemia. While the clinical presentation and laboratory findings initially raised suspicion of myositis, the authors subsequently dismissed this possibility, reasoning that myositis is not recognized as a typical manifestation of TBE.

Hepatic involvement: Elevated levels of transaminases were reported in 11 separate studies [5–8, 10, 14–16, 18–21] involving a total of 112 TBE patients. In 5 distinct studies [7, 8, 14, 15, 18], this elevation exceeded up to 5 times the upper limit of normal, underscoring the varying extent of liver involvement. Moreover, in 2 cases [8, 14], a concomitant elevation in cholestasis parameters was evident. Despite the variability, most cases exhibited transient elevations of transaminases and cholestasis parameters. For example, the study conducted by Misić‐Majerus et al. [7] identified elevated AST and ALT activities in 25 (22%) patients, with the most commonly noted elevation being two‐ to threefold the NVs. Notably, this elevation normalized within 3–4 weeks. Remarkably, the patient described by Hofbauer [14], who had manifest cholestatic hepatitis with histological evidence to support the diagnosis, experienced a complete resolution of the condition, further underlining the potential for favorable outcomes in such scenarios.

Hematological abnormalities: Leukopenia, thrombocytopenia, and, less commonly, neutropenia are commonly reported, reflecting transient bone marrow suppression or immune‐mediated effects. Specifically, leukopenia (WBC < 4.0 × 10^9^/L) was documented in 11 studies involving 183 TBEV patients in total. Thrombocytopenia (defined as a platelet count < 150,000/μm^3^) was reported in eight studies involving 98 TBE patients in total. In the study conducted by Schultze et al. [22], aside from fever, leukopenia and thrombocytopenia were the only clinical manifestations observed in TBEV patients. Additionally, two studies with 91 patients documented neutropenia (defined as an absolute neutrophil count < 1.5 × 109/L). Agranulocytosis, an extreme reduction in granulocytes (defined as an absolute neutrophil count < 0.1 × 109/L), was reported in a single patient in one study [23].

Our patient’s presentation aligns with these observations, revealing that beyond its typical presentation, the disease can also present with various cardiovascular, musculoskeletal, hematological, and hepatological manifestations. First, cardiovascular symptoms encompass a wide range from the occasional transient autonomic dysfunction in the form of reduced heart rate variability and tachycardia [24] to the more uncommon occurrence of manifest myocarditis.

Acute myocarditis is an immune‐mediated inflammation of the heart muscle, primarily driven by lymphocytes. Viral infections (COVID‐19, influenza, parvovirus B19, and HIV) are the most common etiological factors, with less frequent causes being autoimmune conditions (systemic lupus erythematosus) or drug‐induced toxic triggers (especially immune checkpoint inhibitors) [25]. However, the etiology of this disorder remains unknown in most cases. Virus‐mediated myocarditis can be due to “traditional” cardiotropic viruses such as adenoviruses and enteroviruses (especially Coxsackievirus), as well as human herpesvirus‐6 (HHV‐6) and parvovirus B19. Regarding the pathogenesis, it is often unclear whether the myocardial involvement is due to direct infection or an immune‐mediated phenomenon. Since the beginning of the COVID‐19 pandemic, myocarditis has also been recognized as an uncommon but potentially lethal complication of SARS‐CoV‐2 infection, as well as after immunization with mRNA vaccines [26].

A definitive diagnosis of myocarditis can only be made by an endomyocardial biopsy [27]. However, this is an invasive procedure with a high risk of complications and sampling error and is hence infrequently performed in this context. Noninvasive alternatives, such as cardiac MRI and echocardiography, can provide valuable information on myocardial inflammation and function [28]. In our patient, microbiological testing for influenza A, B, respiratory syncytial virus, adenovirus, SARS‐CoV‐2, and enterovirus by PCR from a nasopharyngeal swab in the initial phase of the disease was negative.

In our specific case, the patient presented with fever, chest pain, and elevated cardiac enzyme levels, all while showing no signs of coronary stenosis during coronary angiography. Consequently, the simultaneous occurrence of TBEV infection without evidence of coinfection with other potential pathogens suggested a strong causal correlation. In our case, we did not perform an endomyocardial biopsy to confirm the diagnosis. However, in our opinion, the clinical syndrome together with the positive serology provided strong evidence for acute myocarditis associated with TBEV infection.

Our comprehensive literature review concurred with our findings, highlighting the rarity of myocarditis associated with TBE/TBEV infection and the diagnostic challenges involved. Consistent indicators of myocardial involvement included abnormal echocardiography results and heightened inflammatory markers. In these similar cases documented in the literature, the disease course tended to be benign, ultimately resulting in complete patient recovery.

It is noteworthy to mention that other flaviviruses, such as West Nile virus, dengue virus, Zika virus, and Japanese encephalitis virus, can exhibit comparable symptoms during the acute phase of infection and can also occasionally cause myocarditis, which can be associated with a more ominous prognosis due to potential development of fatal arrhythmia [29, 30].

Second, myositis, characterized by inflammation of voluntary muscles and elevated CK levels, was another intriguing discovery in our analysis. We identified five cases of myositis associated with TBEV infection, each with unique clinical outcomes. Some patients experienced complete recovery, while others faced fatal consequences. Notably, one of these cases involved a pediatric patient, aligning with existing literature that suggests a potential vulnerability of children to viral myositis due to the virus’s affinity for immature muscle cells [31].

Third, elevated liver transaminases were a recurrent finding in our analysis, reported in 11 separate studies. Interestingly, these elevations were often transient and typically returned to normal within a few weeks, even in cases of pronounced liver abnormalities associated with TBEV infections. No case of fatal liver failure was reported to our knowledge.

Lastly, hematological abnormalities—including leukopenia, thrombocytopenia, neutropenia, and even agranulocytosis—emerged as a common theme in TBEV‐infected patients. These often self‐limited hematologic irregularities generally have low clinical relevance but warrant vigilant monitoring given their implications for possible secondary infections and hemorrhagic complications. Their frequency justifies further investigation into the pathophysiological mechanisms by which TBEV disrupts myeloid and lymphoid cell development and homeostasis.

Relating extraneurological manifestations to TBEV remains challenging as these symptoms typically occur early during infection, while seroconversion and detectable IgM/IgG antibodies develop later [32]. Real‐time RT‐PCR for TBEV RNA in cerebrospinal fluid (CSF) and blood can facilitate early diagnosis of TBEV infection [33]. However, as sensitivity rapidly declines once the neurological phase begins, the utility and experience of PCR in clinical practice are very limited. While CSF PCR provides confirmatory evidence of CNS infection in early‐presenting TBE cases, viremia alone rarely persists long enough for PCR testing to remain diagnostically relevant [34]. Overall, clinicians must leverage exposure history and maintain a high index of suspicion to justify early TBEV PCR testing when serological diagnosis remains unreliable.

## 5. Conclusion

In conclusion, our case underscores that myocarditis can be the first manifestation of acute TBEV infection. Extraneurological manifestations of TBEV, which occur mostly during the first phase of the disease, remain relatively uncommon and typically have a benign course with favorable outcomes. However, it is essential to recognize these rare manifestations to prevent potential complications and provide informed patient care. Consequently, clinicians should be aware of a possible cardiac involvement in TBEV in the adequate epidemiological context.

Vaccination is still the most effective means of preventing TBE and is actively encouraged for all people living and traveling to endemic areas.

## Funding

This research did not receive any specific grant from funding agencies in the public, commercial, or not‐for‐profit sectors.

## Ethics Statement

Informed consent was obtained from the patient for the publication of this case report.

## Conflicts of Interest

The authors declare no conflicts of interest.

## Data Availability

The data that support the findings of this study are available upon request from the corresponding author. The data are not publicly available due to privacy or ethical restrictions.
